# Thoracic Ewing’s Sarcoma: A Case Report

**DOI:** 10.7759/cureus.24150

**Published:** 2022-04-14

**Authors:** Akesh Thomas, Nizar Obeidat, Mohammad Darweesh

**Affiliations:** 1 Internal Medicine, East Tennessee State University - Quillen College of Medicine, Johnson City, USA

**Keywords:** thoracic ewing’s sarcoma, ewing’s sarcoma, extraosseous ewing’s sarcoma, askin’s tumor, primitive neuroectodermal tumor

## Abstract

Ewing’s sarcoma family of tumors (ESFTs) contains multiple tumors with similar histological and immunohistochemical features. ESFTs are small, round cell, highly malignant tumors that arise from the neuroectoderm of bone and extraskeletal soft tissue. Ewing’s sarcoma is the second most common primary malignant bone cancer in children and adolescents, with the second decade of life being the most common age of diagnosis. In this article, we present a case of a young male who presented to the emergency department complaining of shortness of breath and cough and was later diagnosed with Ewing’s sarcoma of the chest wall, which is also called Askin’s tumor, and it is an extremely rare disease with only 17 cases reported in the literature.

## Introduction

Ewing’s sarcoma family of tumors (ESFTs) consists of skeletal Ewing’s sarcoma, extraosseous Ewing’s sarcoma, primitive neuroectodermal tumor (PNET), and Askin’s tumor. Ewing’s sarcoma is primarily seen in the second decade of life, with an average incidence of about 2.93 per million population [1]. Ewing’s sarcoma of the thorax, also called Askin’s tumor, is a rare entity classified within PNET by the World Health Organization [2]. Clinically, this often mimics pneumonia with fever, cough, chest pain, and shortness of breath [3]. Due to the rarity of the disease, there are no clear treatment protocols, but a combination of surgical removal of the tumor followed by radiation and combination chemotherapy with two to six drugs, including doxorubicin, actinomycin D, cyclophosphamide, ifosfamide, vincristine, etoposide, busulfan, melphalan, and carboplatin, has shown to improve the prognosis [4]. Here, we report a case of thoracic Ewing’s sarcoma in an 18-year-old male.

## Case presentation

An 18-year-old Caucasian male with no significant past medical history presented to the emergency department complaining of shortness of breath and cough for one month. A week earlier, he was seen in the emergency room for the same complaints and received antibiotics for presumed pneumonia. The cough was described as dry with minimal sputum production. He denied any other associated symptoms. The patient was never a smoker.

**Figure 1:**
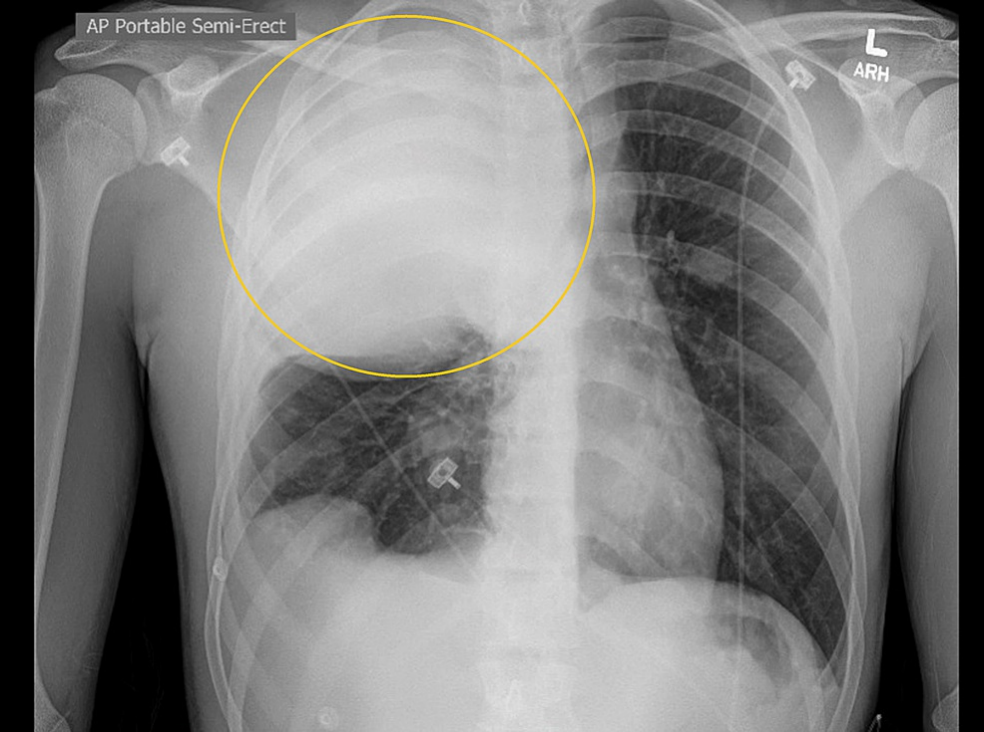
Chest X-ray showing a large mass (yellow circle) in the right hemithorax with a mediastinal and tracheal shift to the left.

**Figure 2:**
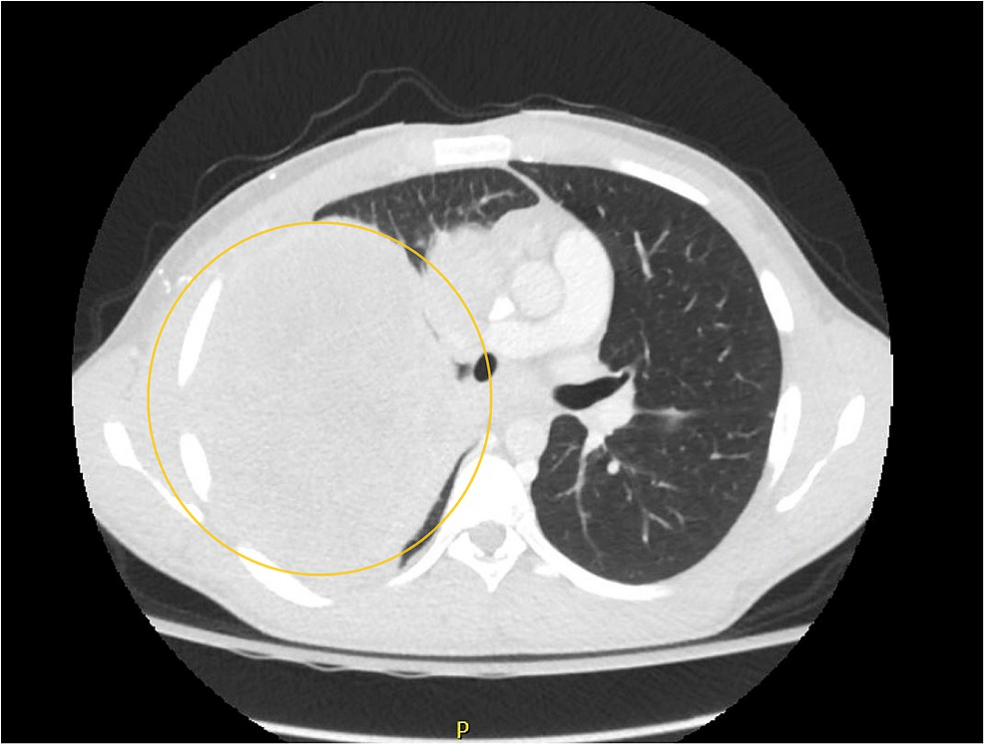
Chest CT scan showing a 15 × 12 × 16 cm mass (yellow circle) extending into the chest wall and right axilla with heterogeneous enhancement and occupying the middle and upper right hemithorax.

**Figure 3:**
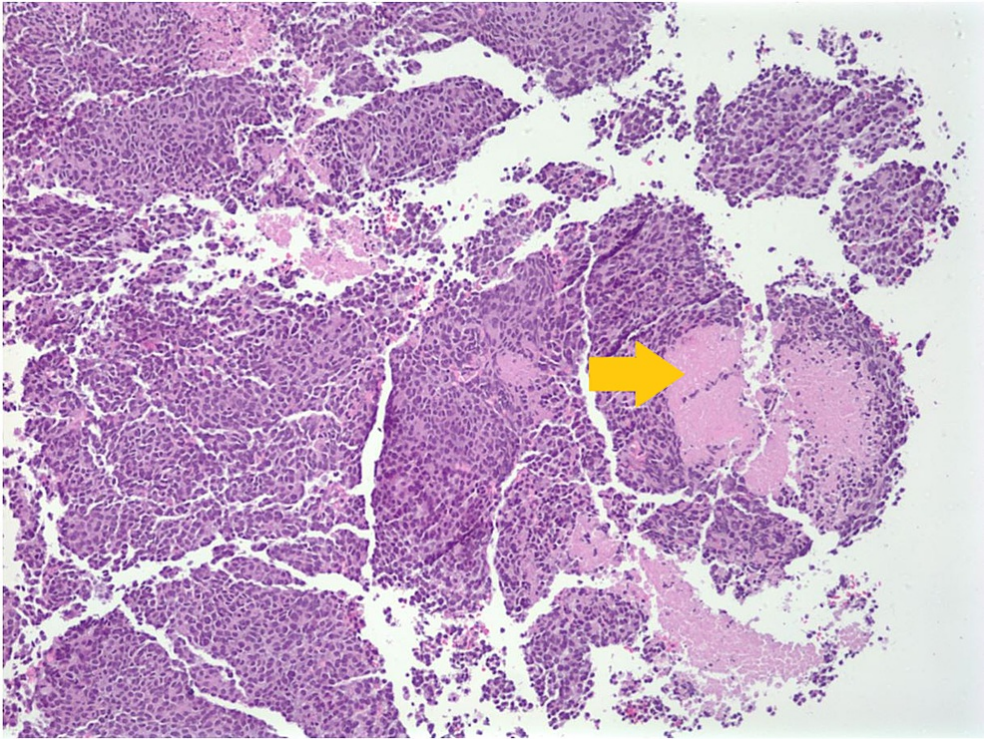
Pathology slide showing areas of tumor necrosis (yellow arrow).

**Figure 4:**
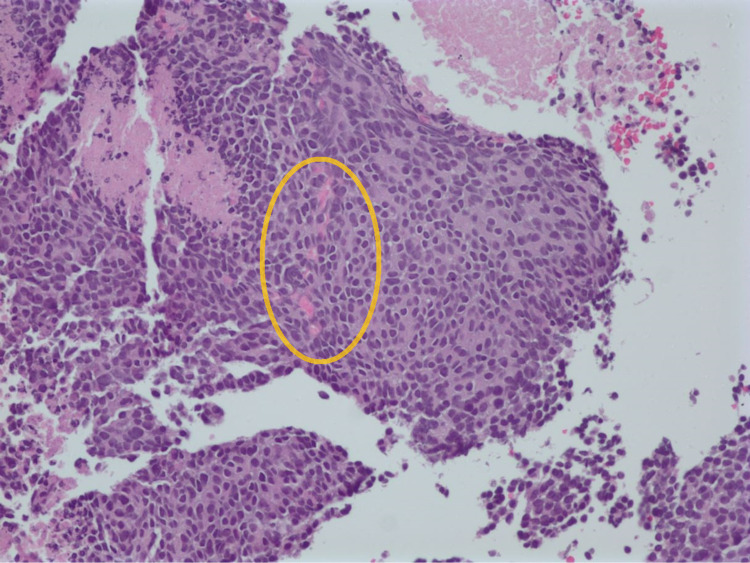
Pathology slide showing small, round, blue cells arranged in a solid pattern with the formation of occasional, vague rosettes (yellow circle).

At the time of presentation, he was tachycardic with a pulse rate of 110 beats per minute and slightly tachypneic with a respiratory rate of 22 per minute and was saturating 98% on room air. The physical examination was significant for reduced air entry on the right side, with bilateral wheeze more prominent on the right side. A chest X-ray obtained (Figure 1) showed a large mass in the right hemithorax with a mediastinal and tracheal shift to the left; this was new compared to the normal chest X-ray about four months before. A CT scan of the chest revealed a 15 × 12 × 16 cm mass, extending into the chest wall and right axilla with heterogeneous enhancement and occupying the middle and upper right hemithorax (Figure 2). The mass was displacing the trachea to the left, with multiple other pleural-based masses. Additionally, a 5.2 × 8.8 cm mass was seen in the right costophrenic angle associated with a small pleural effusion and multiple other small masses on the same side and without evidence of mediastinal adenopathies. Tumor markers AFP, LDH, and B-HCG were negative. A CT-guided biopsy was obtained. The pathological segment (Figures 3 and 4) demonstrated small fragments of tumor tissue composed of a proliferation of small, round, blue cells arranged in a solid pattern with the formation of occasional, vague rosettes. Areas of tumor necrosis were also present. Scant cytoplasm and fine chromatin were noted with infrequent mitotic figures. The immunohistochemistry was positive for CD99 (membranous) and NKX2.2 (nuclear) and was negative for PHOX2B, desmin, CD20, CD45, synaptophysin, chromogranin, GFAP, SALL4, and myogenin. FISH studies detected rearrangement of EWSR1 (22q12) in 60% of the cells, and fusion of EWSR1 and FLI1 (11q24) was detected in about 85 % of the cells. The overall findings were diagnostic of EWSR1-FLI1 fusion Ewing’s sarcoma. A diagnosis of Ewing’s sarcoma was made, and the patient later underwent surgical removal of the mass, followed by radiation therapy.

## Discussion

A cough that persists for more than three weeks and less than eight is considered a subacute cough [5]. Subacute and chronic cough warrant further investigation, and obtaining a chest X-ray is an essential and cost-effective investigation modality. Postinfectious cough, postnasal drip, asthma, gastroesophageal/laryngopharyngeal reflux, and bronchitis are the common causes in the patient’s age group. A detailed history can exclude most of these etiologies. In young adults, thoracic mass can be benign lung or mediastinal tumor, malignant lung or mediastinal tumor, or metastatic tumor. The speed of the development of the tumor is suggestive of malignant etiology in this patient. CT scan confirmed a tumor of the lung with satellite lesions rather than a mediastinal origin. A detailed history and physical examination looking for a potential primary lesion are warranted in cases similar to this. Serum biomarkers are not a reliable diagnostic tool for lung cancers [6]; here, we obtained them primarily to investigate potential metastatic cancer, especially seminoma, considering the patient’s age.

The most common primary lung cancers in adolescents and young adults are blastomas and carcinoid tumors [7]. Primary Ewing’s sarcoma of the lung is exceedingly rare, with only 17 cases identified in the literature [8], but many of them might have been labeled as Askin’s tumors. The incidence of this tumor is greater in Caucasian males, and most of the diagnosed cases are in the third decade of life [9]. For this patient’s chest X-ray, a loculated pleural effusion is a differential for an opaque lesion with a mediastinal shift to the contralateral side. Radiographically, pulmonary Ewing’s sarcoma usually presents a single well-defined mass with an inhomogeneous appearance on chest CT scans. Calcifications and pleural effusions involving the affected side of the chest are seen. Additionally, these masses can extend to the chest wall and mediastinum with invasion and displacement of surrounding structures [10,11]. In our patient, the results of the CT scan of the chest, in this case, go along with what has been reported in the literature for similar cases.

Small, round, blue cells with rosette patterns are typical of neuroendocrine origin tumors, which include the Ewing’s sarcoma family of tumors. Extensive necrosis seen in tissue biopsy is also a feature of Ewing’s sarcoma, and the identification of t(11:22) translocation resulting in the EWS-FLI1 fusion gene is the characteristic feature [12]. EWSR1/FLI1 fusion is seen in approximately 95% of Ewing’s sarcoma and is about 90% specific [13,14]. Ewing’s sarcoma of the chest wall has an optimal prognosis with multimodality treatment, including surgery, radiation, and chemotherapy [15,16].

## Conclusions

Ewing’s sarcoma of the chest wall, also called Askin’s tumor, is an extremely rare disease. It usually presents with cough and shortness of breath like in this case. It is usually misdiagnosed and treated as pneumonia. It should be suspected in young adults presenting with a tumor showing characteristic features. Prompt multimodal treatment is needed for the optimal survival of patients with this tumor.
